# Freshness Assessment and Shelf-Life Prediction for *Seriola dumerili* from Aquaculture Based on the Quality Index Method

**DOI:** 10.3390/molecules24193530

**Published:** 2019-09-29

**Authors:** Jorge Freitas, Paulo Vaz-Pires, José S. Câmara

**Affiliations:** 1 CQM– Centro Química da Madeira, University of Madeira, Campus Universitário da Penteada, 9000-039 Funchal, Portugal; jorge.freitas@staff.uma.pt; 2 ICBAS – Abel Salazar Institute for the Biomedical Sciences, University of Porto, R. Jorge Viterbo Ferreira, 228, 4050-313 Porto, Portugal; vazpires@icbas.up.pt; 3 CIIMAR – Interdisciplinary Centre of Marine and Environmental Research, Terminal de Cruzeiros de Leixões, Av. General Norton de Matos, S/N, 4450-208 Matosinhos, Portugal; 4 Faculty of Exact Sciences and Engineering, University of Madeira, Campus Universitário da Penteada, 9000-039 Funchal, Portugal

**Keywords:** Greater amberjack, *Seriola dumerili*, freshness, shelf-life, QIM, sensory analysis, physicochemical analysis, microbial analysis

## Abstract

Fish and fish-based products are easily perishable foods due to different factors, including fragile organization, abundant endo-enzymes, psychrophilic bacteria, and impact of pre-harvest operations, that contribute to reducing its value. Therefore, a timely effective method for fish freshness and shelf-life evaluation is important. In this context, this study aimed to develop a sensory scheme based on the Quality Index Method (QIM) (sensory table and point system) for freshness monitorization and shelf-life prediction for *Seriola dumerili* from aquaculture in Madeira Island. Evaluation of appearance, texture, eyes, and gills was performed during 20 days of storage on ice (0 ± 1 °C). The shelf-life prediction was supported by the analysis of microorganisms (total viable colonies, TVC, counts), texture (Torrymeter), and production of trimethylamine (TMA), evaluated by HS-SPME–GC–MS and validated according to Association of Official Analytical Chemists AOAC guidelines. The result is a QIM scheme with 25 demerit points, where zero indicates total freshness. From the integration of sensory analysis, microbial growth at the time of rejection (TVC, 10^8^ cfu/cm^2^ and H2S producers, 10^7^ cfu/cm^2^), texture (Torrymeter value < 8), and TMA analyses (>12.5 mg/100 g), shelf-life was estimated as 12 days (±0.5 days). The obtained results show the high-throughput potential of the developed method for fish freshness assessment and shelf-life prediction. This QIM scheme is a secure way to measure quality and provide users with a reliable standardized fish freshness measure.

## 1. Introduction

The European aquaculture sector is dominated by long-established species such as rainbow trout (*Oncorhynchus mykiss*), Atlantic salmon (*Salmo salar*), common carp (*Cyprinus carpio*), gilthead seabream (*Sparus aurata*), and European sea bass (*Dicentrarchus labrax*) [1]. To diversify the product offer, several attempts have been made to introduce new species at the pilot scale or production level in Mediterranean aquaculture [2]. One of the candidates for marine warm water cage culture is *Seriola dumerili* (Risso,1810), because of its high economic value and growth rate (6 kg in 2.5 years) and its diffusion in the circumglobal temperate area [3]. It is also known as greater amberjack (GrA) and by other common names in European territory (Table 1).

In the Mediterranean, GrA production in 2012 was approximately 2 tons, most from wild capture, with hatchery-produced individuals in Malta and some from production efforts in Greece, Spain, Italy, and Cyprus [4]. The conduction of several studies regarding the fatty acid composition of wild and reared fish [5], reproduction [6], feed formulation [7], rearing conditions [8], and handling effects [9] has led to important improvements to overcome some bottlenecks of the European aquaculture industry and GrA aquaculture development. 

Other studies were conducted in terms of consumer acceptability and sensory analysis [2]. Sensory characterization is an important food quality determinant, a driver for consumer acceptance, food choice, and market value. Also, the quality characterization of aquaculture products according to production and post-production factors is always of primordial importance [5,6]. Sensory analysis is adaptable to several points in the supply chain, being relevant to verify fish freshness at different transaction points. Sensory analysis applications will ultimately enhance the overall product perception in the modern demanding markets [7].

At the seafood industrial level, several methods can be used to evaluate the quality of the products to ensure that qualitative parameters are met by all agents in the value chain (producer to final consumer), including regulatory agencies, as well as in different stages of fish processing [8]. It is known that no single method is reliable enough to determine the freshness or quality of seafood products [6]. Recognition of the freshness, and acceptance or rejection, of fish on this basis maybe all that industry needs.

On the other hand, different species and products spoil in different patterns, and the use of appropriate assessment methods is needed. Apart from sensory methodologies, physical, chemical, and microbial methods have been developed [8,9,10]. However, most of them have as fundamental base sensory knowledge to associate the analytical results with overall quality. Therefore, sensory evaluation is still considered one of the most effective techniques to satisfactorily assess freshness and grade fish or fish products [11]. Developed by the Tasmanian Food Research Unit (TFRU), the quality index method (QIM) is a fast, simple, nondestructive, and descriptive, grading system for seafood freshness evaluation. It integrates the differences between fish species through objective assessment of fish attributes (e.g., gills odor) [12]. It also provides the user (producers, buyers, sellers, and resellers) with a reliable and standardized methodology that includes instructions and easily understandable illustrational material. It is well suited to teach inexperienced people and train or monitor panelists’ performance [7]. Attributes are evaluated with a scoring system (0 to 3 demerit points), and as time progresses, higher punctuation is given [6]. However, it is common that one or more attributes do not possess the maximum demerit points because the changes during the storage period may not be significant to achieve such scores [13,14].

After adding up all scores, a quality index (QI) is obtained for shelf-life prediction. The prediction is possible if a linear correlation between QI and storage time is achieved. However, new QIM schemes are constructed with the support of other methods. The evaluation of fish muscle with the Torry Scale [9] is common in regions where fish is mainly commercialized in fillets. For fish sold as a whole, rejection occurs sooner; therefore, it is preferable to evaluate the external characteristics using different methods [13]. Other methods are the measurement of muscle dielectric properties (Torrymeter, TRM), the enumeration of specific spoilage organisms (SSO) [15], and chemical evaluation [9].

The QIM-EUROFISH project has been very important in the dissemination of the QI method, with the publication of the QIM manual in diverse languages, and with 13 QIM schemes as examples [12,16]. A considerable effort to publish and optimize the QIM schemes has been made, demonstrating the increasing importance of QIM for new species and products from wild or farmed species. Information can be found in Barbosa and Vaz-Pires, Bernardi et al., Sant′Ana, Soares, and Vaz-Pires, Ndraha [7,13,17,18], and in the Eurofish-QIM database.The objective of this study was to develop a sensorial scheme based on QIM (sensory table and corresponding point system) for freshness monitorization and shelf-life prediction of GrA, farmed in Madeira Island, through the evaluation of changes in general appearance (skin, anus, and odor), texture (firmness), eyes (color and shape), and gills (color, mucus, and odor) throughout 20 days of storage on ice (0 ± 1 °C). Microbiological analysis (total viable colonies counts, TVCc), physical (TRM) and chemical analyses (pH; and trimethylamine (TMA) quantification through HS-SPME–GC–MS) were also performed.

## 2. Results and Discussion

### 2.1. QIM Development 

**Figure 1:**
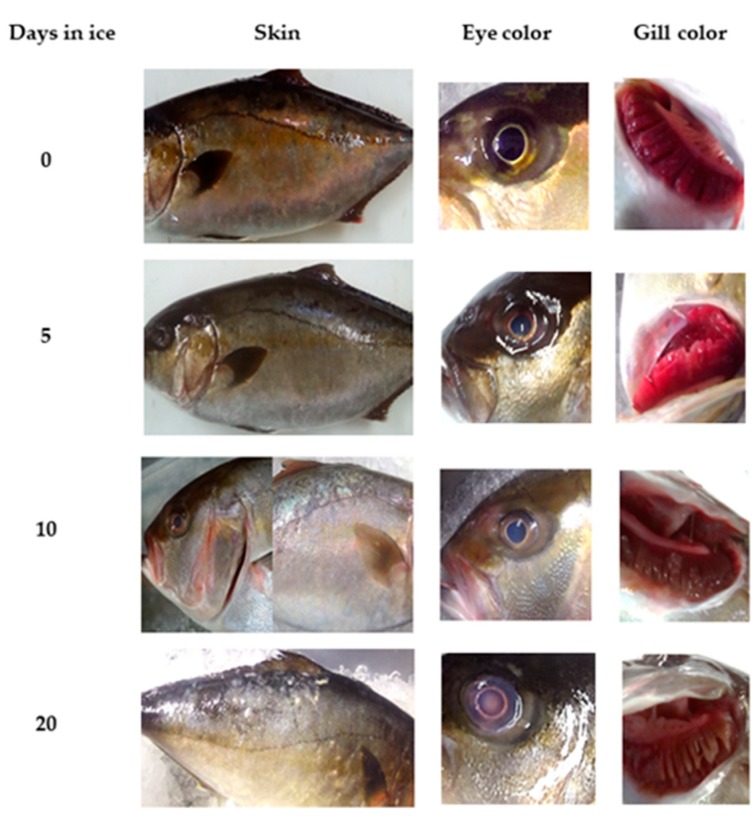
Greater amberjack (GrA) main visual changes during storage at 0 °C on ice.

The parameters that were selected and included in the preliminary QIM were based on the description of external characteristics (skin, texture, odor, appearance of eyes, gills, and anus), as they showed clear changes during the first storage observations (Figure 1). During the development of the preliminary QIM tables, successive modifications were made to obtain the highest possible number of descriptors. 

The panel performance evaluation was conducted by obtaining the QI for each specimen during the second experiment, when the attributes for the evaluation were fixed. A significant correlation (*p* < 0.05) between all assessors’ results was obtained. The slope and r^2^ for each panelist (P) were: P1 (0.9781–0.8945), P2 (1.0083–0.9481), P3 (1.0706–0.9445), P4 (1.0840–0.8510), P5 (1.0754–0.8443), P6 (1.1283–0.8381).

The final scheme suggested in this work includes 25 demerit points, describing four quality attributes with 9 sensory attributes (Table 2).

**Figure 2:**
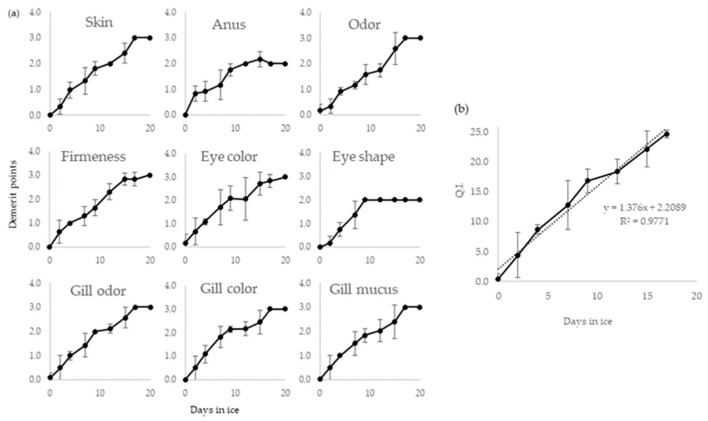
(**a**) Evolution of different QIM parameters for GrA (mean values of the six assessors). (**b**) QIM calibration curve (QI versus days on ice) for GrA during ice storage (0 ± 1 °C). Points that seem without error bars have in fact error bars smaller than the symbol size.

Figure 2 shows the average points registered for each individual attribute as a function of the storage days on ice. It is possible to see that all quality attributes present an increasing trend during storage. QI is the sum of demerit points of the different quality attributes (Figure 2b). The QI for GrA showed a significant linear increase during storage on ice (*p* < 0.01), with a high coefficient of determination (r^2^ = 0.9771) ranging from 0.4 to 25 from day 0 to 17, respectively.

**Figure 3:**
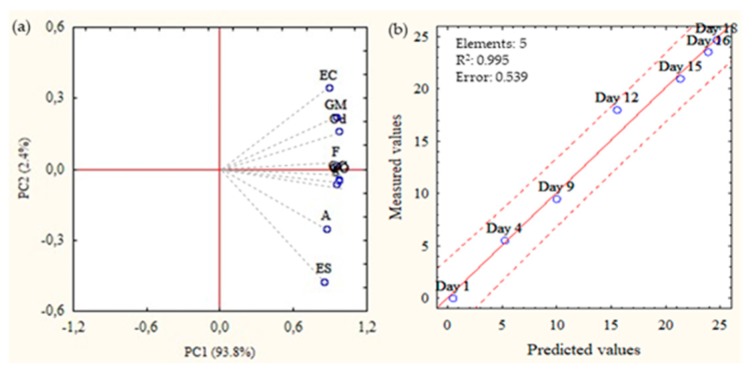
(**a**) Principal component analysis (PCA) correlation plot describing QI values and individual sensory parameters: skin appearance (S), Odor (O), firmness (F), gill color (GC), gill odor (GO), gill mucus (GM), eye color (EC), eye shape (ES), anus (A) of all GrA samples; (**b**) partial least-squares (PLS) regression model of the QI scores of GrA samples versus the predicted values. Trace lines represent the confidence limits of regression (95%).

Principal component analysis (PCA) analysis indicated that the proportion of variance for the first two principal components was 96.2%, PC1-93.8%, and PC2-2.4% (Figure 3a). All variables presented a high positive correlation with the first component; therefore, they contributed with the same approximate weight to QI. Analysis of variable importance confirmed each attribute’s importance. For this scheme, values above 0.8 were considered relevant for the QIM protocol [19]. Thus, the variable importance for each attribute was, in decreasing order: odor–0.959; gill odor–0.958; eye color–0.950; eye shape–0.947; gill mucus–0.946; gill color–0.939; skin–0.919; firmness–0.905, and anus–0.815. However, the correlation results for QI values showed a higher correlation (0.991) than for single attributes. This indicates that the proposed QIM scoring scheme is suitable to follow the deterioration progress and correlate it with time, which is in accordance with other developed QIM methods [20,21].

For the GrA sensory scheme, the odor was one of the main contributors to the determination of deterioration progress. At the start of the trial, the skin and gill odors were described as seaweed or fresh and, from day 3, became neutral. The perception of the off-odors associated with degradation occurred on day 11, but the intensity was only considered sufficient for rejection on day 12. The QI average equations of observed and predicted values were subjected to the partial least-squares (PLS) regression model. The results showed that the obtained linear regression model had an estimation error of 0.54 days and a suitable linear regression (r^2^ = 0.995) (Figure 3b). The predicted and measured values were statistically similar (*p* < 0.05).

The developed QIM scheme successfully described different freshness levels of GrA and sustained individual panelists variation. Taking into consideration all sensory data, the rejection was estimated to occur between days 11 and 12, mainly due to the presence of significant off-odors on the skin and gills (rancid odors).

### 2.2. Microbiological Analysis

Microorganisms associated with aquatic species reflect the bacterial population of the surrounding environment and the conditions during manipulation or processing. In newly processed seafood, SSO exist in low numbers and constitute a small fraction of fish microflora. During storage under particular conditions (e.g., specific temperature), SSO grow faster than the remaining microflora, produce metabolites responsible for off-flavors, and finally cause sensory rejection of a product [9,22]. The most common SSO associated with marine species from temperate waters, stored on ice (0 °C) under aerobic conditions, are *Shewanella spp* and *Pseudomonas spp*. They are associated with ammoniacal spoilt and hydrogen sulfide-type odors [13]. The TVCc after the catch varies between 10^1^ and 10^4^ colony-forming units (cfu)/g or cm^2^, while at times of rejection, the values vary between 10^7^ and 10^9^ cfu/g, according to fish species or fish product [13,14].

**Figure 4:**
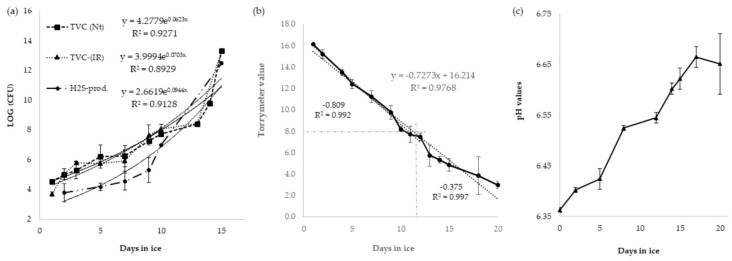
(**a**) Bacterial growth results, shown as total viable counts (TVC) in nutrient agar (Nt) (■); (TVC) in iron agar solid medium (IR) (▲), and H2S-reducing bacteria (●) in ice-stored GrA for 16 days (CFU: colony-forming units). Each point is a mean value of two analyses, with vertical bars indicating standard deviation; (**b**) Torrymeter values of whole GrA during ice storage; (**c**) pH values of GrA samples against storage time on ice. Each point represents the mean values of three analyses, with vertical bars indicating standard deviation.

The microbiological analysis results are presented in Figure 4a. All data shown represent the mean of two different samplings from all individuals tested (*p* < 0.01). Microbiological analysis of the skin showed an initial TVCc of 10^3^ cfu/cm^2^, which increased gradually until day 7 (10^6^ cfu/cm^2^). The H_2_S-producing microorganisms (that appear as black colonies owing to the precipitation of iron sulfide) presented lower TVCc than in nutrient agar (Nt) and iron agar solid medium (IR) during the first 9 days (10^3^–10^5^ cfu/cm^2^). However, after day 9, the plate counts achieved values close to the TVCc in Nt and IR. Using the fitted regressions, the recommended limit values for bacterial counts (10^7^–10^8^ cfu/cm^2^) was surpassed after 10 to 11 days, with values of 10^8^ cfu/cm^2^ for TVCc on Nt and IR, while for H_2_S producers, the plate count was 10^7^ cfu/cm^2^. Counts of *Enterobacteriaceae* on MacConKey agar (McA) were very low, never exceeding 10^2^ cfu/cm^2^ or being negligible. The general hygiene condition of fish samples depends on water quality and handling, which starts on board, and seemed to be acceptable for the samples studied, taking into account the results obtained in this work.

### 2.3. Physical Evaluation

The base for electrical measurements on fish is the fact that the cell membranes in fish muscle tissue are progressively disrupted by autolytic enzymatic degradation and, later, by microbial action, leading to a decrease of electrical resistance and capacity. Therefore, as time progresses, the amount of electric impulse detected will be lower [22]. During the three storage experiments, all fishes were analyzed during harvesting, handling, and storage in order to reduce the variability of equipment readings due to differences in biological condition and physical damage to the tissues. They could present lower or more variable dielectric properties, indicating apparently advanced spoilage [20]. 

The changes in dielectric properties during storage on ice of the GrA samples are shown in Figure 4b, which shows the mean of all fishes from the three trials. At the beginning of storage, the TRM means values (>16) of GrA samples indicated “very fresh fish”, and a relatively high negative correlation with storage time was observed (r^2^ = 0.9768).

The mean TRM values decreased almost linearly in the course of the first 11 days of the trial (16.0–8.0). Between 12 and 20 days, TRM values varied less (from 8.0 to 3.0), creating two distinct regions with different slopes. The first region showed a slope value of −0.809 (r^2^ = 0.992), and the second region presented a value of −0.375 (r^2^ = 0.997). The change in slope is a consequence of the two-phase quality-loss phenomenon explained by two different mechanisms of degradation, i.e., enzymatic autolysis and microbial action [23]. The change of the trend of the slope could indicate the mean value for consumption acceptability [23]. In this study, the slope change occurred between the mean values of 7 and 8, corresponding to 11–12 days of storage. The value of 8 as the limit for consumption is also used for other species [24,25].

The proposed value for consumption acceptability is also in agreement with the QIM values, which set sensory rejection between 11 and 12 days of storage. The TRM values were interpreted as follows: absolutely fresh fish was given a score of 16; fish in the commercially useful storage period received a score of 8 or more; scores below 7.5 would be indicative of low-quality products for consumption. The results presented here show that this instrument is useful for quality evaluation during the first 11–12 days of storage. 

### 2.4. pH Analysis

The determination of the concentration of hydrogen ions (pH measurement) in fish products can be used to assess the degradation progress [19]. The typical pH of live fish varies between 7.0 and 7.3. After death, the pH is generally between 6.0 and 6.8 and in some species, is below 6.0, because of high glycogen concentrations. The results of the pH measurements during spoilage showed that after rigor mortis, the pH increased from 6.4 to 6.7 (Figure 4c), throughout the experiment duration. However, pH measurements were not statistically significant (*p* = 0.21) and may not be suitable for GrA, in contrast to other species, because of individual initial differences, fluctuations of the values, and the low correlation with the sensory results [21,22]. 

### 2.5. TMA Analysis

#### 2.5.1. HS-SPME–GC–MS Method Validation 

HS-SPME, developed for the analysis of volatile compounds, has demonstrated a unique capability of incorporating extraction and concentration in one single step as well as compatibility with GC–MS [26,27]. The validation of the analytical methodology for TMA analysis is presented in Table 3.

Calibration curve linearity was calculated as suggested by Araujo, 2009 [28]. The experimental Fisher value was compared to the critical theoretical Fisher value at 95%, with 12 and 4 freedom degrees (3.26). If the experimental dataset describes a proposed function calibration of the given form, then the condition (F_theo_. > F_exp_.) must be fulfilled. The best result was achieved for the presented 2nd degree polynomial equation. The results obtained for the remaining evaluated parameters were in agreement with the reference values stipulated by the AOAC guidelines [29]. For the matrix effect, the reference is between 70 and 125%; the intermediate precision and repeatability references are below 15% RSD, and recovery is 80–115%.

#### 2.5.2. Method Application for TMA Quantification in GrA

**Figure 5:**
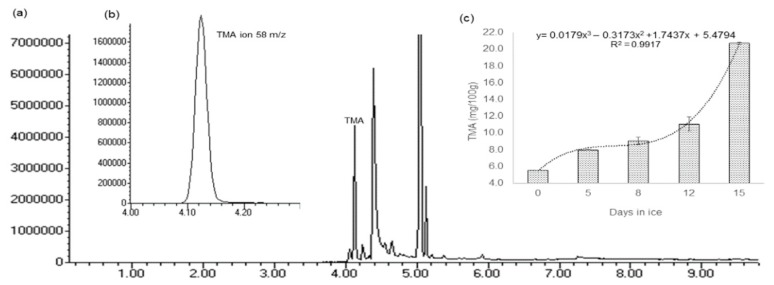
(**a**) Total ion chromatogram of a GrA sample obtained through the HS-SPME/GC–MS method. (**b**) TMA selected-ion chromatogram 58 *m*/*z*, from the GrA sample. (**c**) Results of TMA quantification with the HS-SPME/GC–MS method, during 15 storage days. Each bar represents the mean of three analyses, with vertical bars denoting standard deviation. Points that seem without error bars, have, in fact, error bars smaller than the symbol size.

TMA is a volatile amine, commonly associated with the typical “fishy” smell during fish degradation. It can be produced by the bacterial reduction of trimethylamine oxide (TMAO) in marine fish species [30]. A small production of volatile nitrogen components could indicate limited growth of bacteria that produce TMA, such as *Shewanella putrefaciens* [21]. In Europe, the limit established for TMA, according to Directive 91/493/EEC, is 12 mg/100 g [31]. HS-SPME developed for the analysis of volatile compounds, has demonstrated the unique capability of performing extraction and concentration in one single step and compatibility with GC–MS [26]. The results obtained from the application of HS-SPME/GC-Ms are presented in Figure 5a,b.

The obtained data indicated a gradual slow increase of TMA content during the first 7 days (5.6–9.0 mg/100mg) (Figure 5c). Between 12 and 15 days, the TMA content increased much faster, since the values went from 11.1 to 20.7 mg/100 mg during this period. This is in accordance with the microbiological results obtained in this work. Using the fitted model for TMA data, the 12 mg/100 mg criteria could be reached between 12 and 13 days.

Since the fish deterioration process involves several simultaneous changes, Table 4 summarizes the results for sensory, physicochemical, and microbiological evaluation, corresponding to the criteria used to predict and estimate GrA shelf life.

## 3. Materials and Methods 

### 3.1. Samples

Fresh GrA specimens were acquired from Ilhapeixe SA (IP) aquaculture facilities in Ribeira Brava, Madeira island, Portugal. A total number of 30 specimens were captured between August and September 2018. The fishes were killed in a mix of ice and saltwater, placed on ice, and transported in polystyrene boxes to the company’s refrigerated facilities. The time between capture and arrival at the facilities was between 3 and 4 h. The fishes where washed, placed in perforated polystyrene boxes, and conserved on crushed ice at 0 °C (±1 °C) for a maximum period of 20 days. Fresh ice was added when necessary. The samples had an average weight between 1.5 and 2.5 kg and a total length between 51 and 66 cm.

### 3.2. Sensory Analysis, QIM

The QIM protocol was carried out as reported by several authors and the method reference manual [7,13,16,22]. It consisted of three stages, two training sessions, and one validation step. To develop the quality index for ice-boxed GrA, six trained QIM assessors were selected among the staff of the fish factory IP and the CQM-Centro de Química da Madeira, on the basis of previous experience with the fundamentals of whole-fish sensory analysis, no natural impediments (e.g., allergies or low sensory sensibility), non-smoker status, vocabulary usage, and knowledge of the studied fish species. The first trial was necessary to familiarize the assessors with the degraded fish characteristics, find the parameters that change with time, and obtain a first draft of the QIM scheme. The QIM scheme for GrA was established considering attributes for appearance, texture, eyes, and gills and descriptions of how they change with storage time (0, 2, 4, 7, 9, 12, 15, and 20 days). Scores, ranging from 0 to 3, were given to each key attribute according to the descriptions, being 0 totally fresh fish, and 3 clearly spoiled fish. For attributes that do not present four clear degradation stages, the score was attributed according to the stage that was clearly present when the fish was rejected (i.e., eyes having three clear alterations (0, 1, and 2) during the period of the trials. Since a fourth alteration was not detected, the score 3 was not attributed).

The second experiment was used to confirm the first trial impressions, clarify points that were less clear, and retrain the assessors with the obtained QIM scheme (1, 3, 6, 10, 14, 17, and 20 days). The panel performance was evaluated by obtaining the QI for each participant during these experiments. The parameter evaluated were QI slope and r^2^ values of the panelists.

The third experiment was performed using the developed QIM scheme, and the assessors were unaware of the storage time (0, 2, 4, 7, 9, 12, 15, 17, and 20 days). The samples of each batch were presented to the panel in random order. All observations of GrA were conducted under standardized conditions at room temperature. A minimum of three fishes per lot was evaluated to reduce natural variations effects. The panel performance was evaluated by obtaining the QI for each participant during the three experiments.

### 3.3. Microbiological Analysis

Microbiological analysis was performed according to Sant’Ana et al. [13]. Areas of 15 cm^2^ of fish skin were sampled with sterile cotton swabs, and 1 ml of cooled ¼ Ringer solution (Oxoid, Basingstoke, Hampshire, UK) was used for microorganism transfer. After serial decimal dilutions, inoculation was made using the 20 µL drop method on IR, Nt (both from Oxoid, Basingstoke, Hampshire, United Kingdom), and McA (PanReac AppliChem, Darmstadt, Germany). TVCc, as well as selective counts of H2S-producing bacteria, were performed after 48h of incubation at room temperature (23 °C). *Enterobacteriaceae* plates were grown at 30 °C and counted after 3 days of incubation. Duplicate counts were performed at day 1, 2, 3, 5, 7, 9, 10, 13, 14, 15 and expressed as log cfu/cm^2^. 

### 3.4. Physical Evaluation 

The physical evaluation was executed with the TRM 295 (Distell, West Lothian, Scotland, UK) and performed according to the equipment user manual [24]. The fish anterior-dorsal area was chosen for measurements, both sides were analyzed, and residual ice was cleared from the surface. The electrodes were cleaned between measurements and placed on ice to maintain a temperature similar to that of the fish (around 0 °C). All fishes from the trials were tested at days 1, 2, 4, 5, 7, 9, 10, 11, 12, 13, 14, 15, 18, and 20 of storage.

### 3.5. pH Measurement 

The pH measurements were carried out on a 5:1 ratio of distilled water/fish homogenate, using a glass electrode at 20 °C according to Kyrana et al. [32]. The pH of distilled water, controlled before use, was between 7–7.4. Analyses were performed at 0, 2, 5, 8, 12, 14, 15, 17, and 20 days.

### 3.6. TMA Analysis

#### 3.6.1. HS-SPME Procedure 

TMA was isolated through the HS-SPME method. Sample preparation was based on previous works, with minor alterations [33]. The fish flesh was blended in trichloroacetic acid (TCA 7.5%) in a 1:2 (w/w) ratio. After centrifugation at 10,000× *g* for 10min at 4 °C, the supernatant was stored until analysis (−80 °C). The SPME procedure was carried out in accordance with the previously described high-throughput extraction protocol, with slight modifications [34]. Briefly, 0.5 mL of supernatant, 1 mL of a 15 M NaOH solution, and 1 mL of saturated NaCl solution (35%) were added to an 8 mL SPME glass vial. Propylamine was used as an internal standard. The vial was then placed in a 35 °C water bath, and a divinylbenzene/carboxen/polydimethylsiloxane (DVB/CAR/PDMS) fiber was exposed into the headspace for 30 min. After this period, the fiber was withdrawn and injected into the injection port of the GC–MS equipment (Agilent Technologies 6890N; Palo Alto, CA, USA) for analysis.

#### 3.6.2. Method Validation

The following parameters were chosen for method validation: calibration function and linearity, precision, accuracy, limits of detection (LOD) and quantitation (LOQ), matrix effect according to Araujo, Silva et al., [28,35], and AOAC guidelines [29]. In brief, the calibration function was performed with six different TMA concentration points in the 0.1–15 µg/mL range. F-test was used to evaluate linearity, checking the suitability of the function model. The precision was determined by repeatability and intermediate precision, measuring inter- and intraday variation, respectively. The method of “standard additions” was used to evaluate the matrix effect. The LOD and LOQ determination was based on the standard deviation of the calibration curve interception and the slope of a regression curve.

#### 3.6.3. GC–MS Conditions.

The GC–MS analysis was performed with an Agilent BP-20 (30 m × 0.25 mmi.d. × 0.25 μm film thickness) column. The inlet initial temperature for TMA desorption was 235 °C. The run conditions were: initial temperature 80 °C (2 min hold), ramped to 220 °C (50 °C min^−1^), and kept for 5 min. The total run time was 9.80 min. Carrier gas flow (helium) was set at 1.0 mL min^−1^ under splitless mode. The electron impact (EI) mode was 70 eV. TMA analysis was performed through specific ion selection (58 *m*/*z*). TMA identification was accomplished through interpretation of spectra and matching using the Agilent MS ChemStation Software, equipped with an NIST05 mass spectral library, and by comparison with commercially standard. 

### 3.7. Statistical Analysis

The data statistical treatment was performed using the software STATISTICA 10.0 (Stat Soft, Inc., Tulsa, OK, USA). Analysis of the association between sensory variables included in the QIM scheme was performed with PCA. Error Estimation of the QIM scheme was carried out by PLS regression.

## 4. Conclusions

QIM methodologies are being widely applied to various fish species [36,37,38,39] and products [40,41], reflecting the importance of the methodology for fish freshness evaluation and shelf-life prediction. Besides the expected differences between different species, it is also possible to obtain variations in the values of the evaluation parameters for the same fish species, mainly due to storage conditions or type of product [42,43,44,45], reflecting the expected variance that other methods do not incorporate (i.e., EU scheme).

To the authors′ knowledge, this is the first QIM scheme developed for GrA. A quality index with a maximum of 25 demerit points and 9 descriptive parameters (skin and anus appearance; fish odor; firmness; eye color and shape; gills mucus, color, and odor) is proposed. By applying this QIM scheme on whole, ungutted GrA, it was possible to estimate a shelf life of 12 days (±0.5) on ice. 

As stipulated in the QIM methodology, the results were compared with those of other freshness-evaluation methods, such as dielectric properties determined by TRM, TMA quantification, and microbiological analysis. From these comparisons, it was possible to confirm the shelf-life prediction. The results of TRM reflected the textural alterations of GrA during the storage period, starting with a maximum value of 16 and a rejection value of 8 between days 11 and 12, when off-odors were also detected. In other studies, the value 8 was also reached when rejection was determined [24,25]. The microbiological analysis also showed a significant increase of bacteria during ice storage. TVC values reached the rejection limit (between 10^7^ and 10^8^ cfu/cm^2^), after 10–11 days. The results are similar to those of other studies that also correlate microbial rejection values with sensory rejection time [13,17,21,46]. TMA quantification through HS-SPME–GC–MS was compared with quantification by reference methods, such as total volatile base nitrogen (TVBN) quantification, and good correlations were achieved [33,34,47]. In the present work, the TMA rejection value (12 mg/100 g) was reached between 12 and 13 days. TMA production is also related to the increase of microbial load on fish, so it is common that the TMA limit is achieved when SSO are present above the rejection limits [30].

Even though newly developed methods for identifying and measuring spoilage parameters have been developed [8], they are used so far almost exclusively in research laboratories or, to some extent, in projects in cooperation with the industry [22]. Both classical and instrumental verification of freshness assurance require fast inexpensive procedures, and their results must correlate with those of sensory analysis to be useful in quality control. The methods used in daily practice in small and medium enterprises (the majority of seafood enterprises, as for example IP) are those which have been used for decades (TVB-N, pH, sensory inspection) and do not require extensive economic effort to be implemented or maintained [22]. The developed QIM scheme offers detailed information about sensory quality, fulfilling, primary producer’s knowledge, and requirements to integrate traceability systems, without the need for higher investments. Further validation of the scheme, under different seasons, storage conditions, or handling procedures may be required for the future application of the proposed scheme. However, the strengths of QIM indicate that it has high potential to become a reference method in Europe. 

## Figures and Tables

**Table 1:** Common names for *Seriola dumerili* by country.

Country	Common Name
Germany	Bernstein-Stachelmakrele; Bernsteinfisch; Grünel
Spain	Cèrvia; Pez de limón; Seriola; Serviola
UK	Amberjack; Greater amberjack
France	Ciriola; Seriole
Portugal	Charuteiro; Charuteiro-catarino; Írio; Lírio
Croatia	Bilizmuša; Felun; Gof; Orhan; Orva
Malta	Accola; Cervjola; Serjola; Serra
Denmark	Stor ravfisk
Sweden	Bärnstensfisk; Seriola
Serbia	Orfan; Orhan
Slovenia	Gof
Italy	Acciola; Alice grande; Alici; Alicosa; Aricciola; Cavagnola; Jarrupe; Lampuga; Lecc; Leccia; Lecciutte; Licciòla; Lissa; Lissa bastarda; Lupina; Occhio grasso; Ricciola; Sartaleone; Seriola; Seriola di Dumeril; Sirviola; Sumu
Greece	Magiatiko; Manali; Mayàtico; Mayatiko
Finland	Isopiikkimakrilli

**Table 2:** Quality index method (QIM) scheme proposed for *S. dumerili* containing a description for each parameter and the attributed scores (from 0 to 3).

Attribute	Parameter	Description	
**Appearance**	**Skin**	Very bright and iridescent; Side brownish (>50% covered)	0
		Bright; side brownish (<50% covered)	1
		Pale and dull; Side greyish; mouth or operculum reddish-pink	2
		Marked; Superficial mucus; Blood on operculum and mouth	3
	**Anus**	Clean and closed	0
		Abundant yellow feces	1
		Dry light brown feces and open	2
	**Odor**	Sea, seaweed	0
		Neutral	1
		Rancid; Metallic	2
		Putrid	3
**Texture**	**Firmness**	In rigor mortis	0
		Firm, recovers shape fast (≤2 sec)	1
		Softness; recovers shape slow (>2 sec)	2
		Clearly marked and spongy	3
**Eyes**	**Color**	Black; shinny	0
		Black opaque/slightly milky	1
		Greyish/milky	2
		White/grey in partial or total eye	3
	**Shape**	Convex	0
		Flat	1
		Concave	2
**Gills**	**Color**	Dark red	0
		Light red	1
		Brownish red	2
		Brown, grey, or discolored; Bacteria present	3
	**Mucus**	Absent	0
		Present and colorless	1
		Whitish/cream	2
		Abundant, brown, or yellowish	3
	**Odor**	Sea or seaweed	0
		Neutral	1
		Rancid; metallic	2
		Putrid	3
**Total Demerit Points**			**0–25**

**Table 3:** Summary of trimethylamine (TMA) method validation parameters.

Validation Parameters ^1^	
Concentration range (µg/mL)	0.1–15
Regression equation	y = −0.0108x^2^ + 1.080x + 0.1102
R^2^	0.9994
Linearity test (F_theo_/F_exp_)	2.51 ^2^
LOD (µg/ml)	0.6
LOQ (µg/ml)	1.9
Matrix effect (%)	106
Recovery (%)	93.3 LL ^3^; 98.0 ML ^4^; 99.0 HL ^5^
Intermediate precision (% RSD)	8.4 LL; 7.1 ML; 9.7 HL
Repeatability (% RSD)	8.2 LL; 6.0 ML; 9.6 HL

^1^ For method validation, triplicate measurements were taken; ^2^ *p*-value calculated experimentally through *t*-Student test between slopes obtained from the calibration curves for TMA in 7.5% TCA and TMA in fish samples. If *p*-value ≥ 0.05, no significant matrix effect was observed. ^3^ LL: low-level concentration of TMA; ^4^ ML: medium-level concentration of TMA; ^5^ HL: high-level concentration of TMA.LOD: Limit of detection; LOQ: Limit of quantification; RSD: Relative standard deviation.

**Table 4:** Rejection values and estimated rejection day according to each assay.

Method	Rejection Criteria	Value at Rejection	Estimated Rejection Day
QIM	Off-odors	17.5	11–12
Torrymeter	Slope change	8	11–12
Microbiology (TVC)	Log (cfu/cm^2^) = 7–9	8.0	10–11
Microbiology (H2S)	Log (cfu/cm^2^) = 7–9	7.5	10–11
Trimethylamine (TMA)	12 mg/100g	12.5	12–13
